# Optimization of Dengue Immunoassay by Label-Free Interferometric Optical Detection Method

**DOI:** 10.3390/s140406695

**Published:** 2014-04-10

**Authors:** María F. Laguna, Miguel Holgado, Francisco J. Sanza, Alvaro Lavín, Ana López, Rafael Casquel

**Affiliations:** 1 Group of Micro-nano Photonics and Biophotonics, Laser Center, and Center for Biomedical Technology, Universidad Politécnica de Madrid, 28040 Madrid, Spain; E-Mails: m.holgado@upm.es (M.H.); fj.sanza@upm.es (F.J.S.); alavin@etsii.upm.es (A.L.); ana.lopez@upm.es (A.L.); Rafael.casquel@upm.es (R.C.); 2 Applied Physics Department, Escuela Técnica Superior de Ingenieros Industriales, Universidad Politécnica de Madrid, José Gutiérrez Abascal, 2, 28006 Madrid, Spain

**Keywords:** photonic sensing cells, biosensors, label-free immunoassay, dengue virus

## Abstract

In this communication we report a direct immunoassay for detecting dengue virus by means of a label-free interferometric optical detection method. We also demonstrate how we can optimize this sensing response by adding a blocking step able to significantly enhance the optical sensing response. The blocking reagent used for this optimization is a dry milk diluted in phosphate buffered saline. The recognition curve of dengue virus over the proposed surface sensor demonstrates the capacity of this method to be applied in Point of Care technology.

## 1. Introduction

Dengue is an endemic viral disease affecting the human population in tropical and subtropical regions around the world. The number of dengue virus cases has grown dramatically in recent decades: 2.5 billion people in more than 100 countries are at risk of acquiring dengue viral infection. There are more than 50 million new infections annually, with a number of deaths ranging between 20,000 and 25,000, mainly in children [1].

Dengue is an arthropod-borne flavivirus that comprises four distinct serotypes (DEN-1, DEN-2, DEN-3 and DEN-4) that constitute an antigenic complex of the genus flavivirus, family Flaviviridae. The infections can result in a range of clinical manifestations from asymptomatic infection to dengue fever (DF) and the severe disease dengue hemorrhagic fever/dengue shock syndrome (DHF/DSS).

The conventional techniques for dengue detection such as virus isolation, nucleic acid or antigen detection test involve expensive complex procedures [2,3] and serological tests are more indirect, easy to use, but a compromise on sensitivity and specificity [4].

Currently, immunoassay based Enzyme linked ImmunoSorbent Assay (ELISA) tests are commercially available for qualitative or semi-quantitative measurement of dengue virus [5] and they are more accurate, sensitive and specific tests but a significant amount of antibodies/reagents is required. The new techniques for dengue detection have been focused towards development of an effective, accurate and inexpensive device [6,7].

In this work we report an optimization of a direct immunoassay for dengue virus detection employing a recent label-free interferometric optical detection method [8]. This optical biosensing technique permitted us to evaluate and optimize the immobilization of the anti-dengue bioreceptors and the blocking steps in order to be capable of effectively detecting this dengue antigen by an efficient, reliable label-free method, which is much more cost-effective than labeled alternatives and other label-free alternatives.

## 2. Experimental Section

### 2.1. Biosensor

For this experimental work we used as photonic transducer a Biophotonic Sensing Cell (BICELL) [8–10] based on Fabry-Perot interferometers of a square size of 1 cm^2^ and on SU8 polymeric thin film that exhibits a reliable optical label-free biosensing capability. For the BICELL fabrication we use SU8 2000.5 (MicroChem Corp., Newton, MA, USA) diluted 1:10 in cyclopentanone; this SU8 was deposited by spinning and then soft-baked at 70 °C for 1 min. An exposure to UV light process was then carried out, followed by a post-bake step at 70 °C for 5 min to give a stable thin film. Finally, an acid catalysis step was carried out in order to increase the hydrophilicity of the film. The label-free optical biosensing is carried out by monitoring the changes in the optical mode response provoked by the occurence of both the immobilization of the anti-dengue bioreceptors and the recognition of dengue virus antigen.

### 2.2. Optical Characterization of the Biosensor and Sensing Principle

Optical read-out of the biosensor is based on the Interferometric Optical Detection Method (IODM), an advantageous optical label-free biosensing interferometric read-out. This IODM is characterized by the use of two interferometric signals, which allows for the optical reading system to convert the changes caused by the optical transduction into a unique, sensitive variable of detection [8]. Thus, by means of two BICELLs, one employed as interferometric reference and other used for monitoring the presence of biomolecules, we optically characterized the biosensing response. We employed a Bruker Vertex 70 (Bruker Optik GmbH: Bremen, Germany) Fourier Transform Visible and Infrared spectrometer (FT-VIS-NIR): adapted for visible and near infrared spectral range choosing the proper spectral range to optimize the biosensing response and improve the LoD of the system. We followed the well described procedure very recently reported in the literature [8]. Experiments were carried out at a controlled temperature of 20 ± 1 °C.

### 2.3. Immunoassay Procedure

The Ab-dengue/dengue direct immunoassay (from Vircell, Granada, Spain) was carried out on the SU8 polymeric sensing surface of the BICELLs, allowing label free detection by the accumulation or recognition of biomolecules. Since it is important to avoid nonspecific adsorption, the biosensing response was tested against two types of blocking agents for this immunoassay from Vircell: 5% BSA and 5% skim milk. Incubating the antibody dengue onto the sensing surface until saturation permitted carrying out the recognition experiments and assessment of the blocking step performance. Thus, in a second phase, the two types blocking agents were studied.

### 2.4. Dengue Virus Recognition

After the blocking step we proceeded to make a direct immunoassay with the samples of dengue virus without purification containing other types of proteins and biomolecules provided by Vircell.

## 3. Results and Discussion

**Figure 1.**
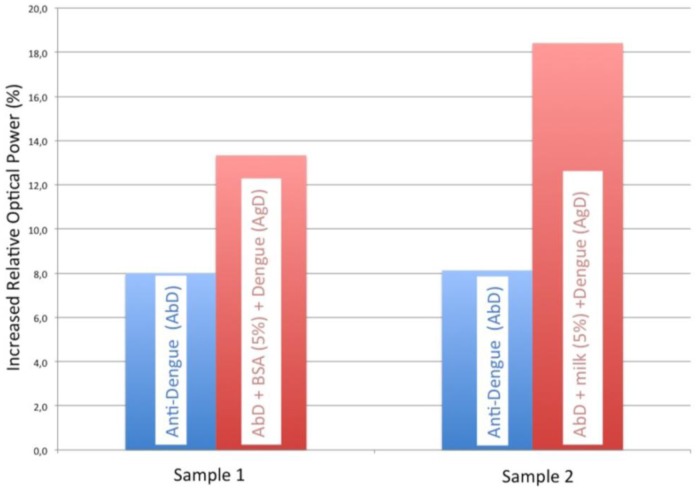
Comparison of two different blocking reagents for detecting dengue virus: BSA (5%) and skim milk (5%).

Several steps where carried for the optimization of the dengue immunoassay: we firstly incubated the dengue antibody onto the sensor surface until saturation, and then we performed and studied the blocking agent to avoid nonspecific adsorption to optimize the immunoassay. The blocking agents studied were BSA and skim milk, being the latter the one which offered a better response (see Figure 1).

The study of the two types of blocking agents on the proposed sensor allowed us to select the most suitable one for our immunoassay. In Figure 1 the blue signal corresponds to dengue antibody immobilization and the red signal is the blocking agent plus dengue antibody. It can be observed that the 5% skim milk signal is greater than the corresponding BSA signal. Therefore, the use of skim milk as the blocking agent achieves a better sensitivity and specificity in the antigen-antibody reaction of virus dengue for an optimal detection. In fact, for the same anti-dengue signal, the dengue recognition increases with a skim milk blocking step.

Since 5% skim milk was the most suitable blocking agent for our immunoassay we studied the response of dengue virus without purification over the surface sensor with and without blocking agent.

**Figure 2.**
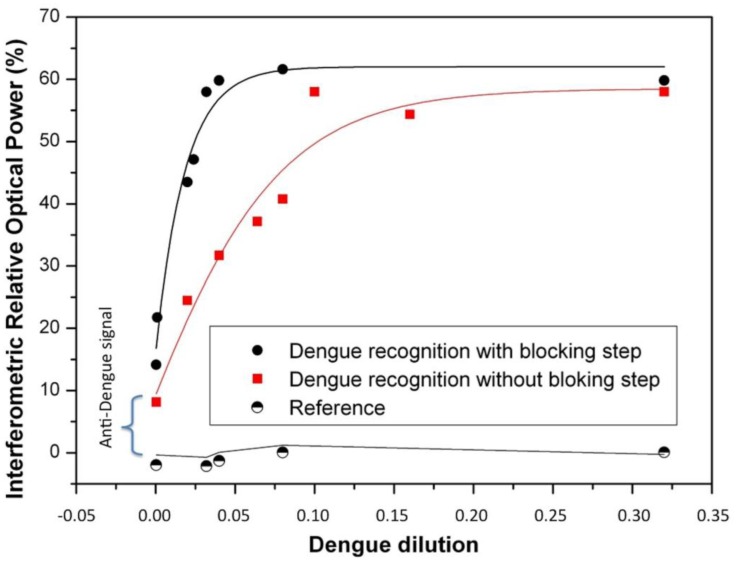
Comparison of sensing curves for dengue recognition with and without blocking step, where the skim milk reagent significantly improves the biosensing response.

Thus, after selecting the most suitable blocking agent, a performance comparison with and without blocking agent was carried out. Figure 2 shows these recognition curves of dengue virus with and without blocking agent. The reference signal is a BICELL without Dengue antibody. The biosensing dengue virus recognition response was significantly improved using the selected specific blocking agent (5% skim milk). In fact, the sensitivity defined as the slope of the transducing signal (Increased Relative Optical Power) as a function of the antigen concentration, was 2.5 times better in comparison with the recognition without any blocking step. We attribute this to the fact that skim milk was the best agent to block the sites and avoid the adsorption of non-specific components onto the sensing surface.

## 4. Conclusions

We have developed a sensor based on SU8 polymer able to detect dengue virus through an optical label-free biosensing technique. The optical read-out for the characterization of the biosensor is based on the Interferometric Optical Detection Method. We efficiently optimized the dengue immunoassay by means of an appropriate blocking agent (5% skim milk), thus avoiding non-specific absorption, and therefore obtaining a high affinity reaction between antigen and antibody.

The response curve of the dengue Virus shows that the proposed sensing system it is a good alternative for cost-effective *in-vitro* dengue diagnostis as a good approach for optical Point of Care technologies. Moreover, with this biosensing systems a competitive limit of detection can be achieved in comparison with other optical label free biosensors [7,8]. In fact, from a cost perspective, the BICELLs-based optical biosensor is scalable at the wafer level ensuring mass production in a cost-effective manner. Moreover, the IODM does not need costly optical dispersive elements such as (wavelength) gratings for the read-out, implying a lower cost for the biosensors optical readers.
